# Ion-Selective Electrode Based on a Novel Biomimetic Nicotinamide Compound for Phosphate Ion Sensor

**DOI:** 10.3390/polym14163392

**Published:** 2022-08-19

**Authors:** Bongjin Jeong, Jin Seong Oh, Do Yeob Kim, Dong Gyu Kim, Young Il Kim, Jungseok Heo, Hyung-Kun Lee

**Affiliations:** 1 ICT Creative Research Laboratory, Electronics & Telecommunications Research Institute, Daejeon 34129, Korea; 2 Department of Chemistry, Chungnam National University, Daejeon 34134, Korea

**Keywords:** ion-selective electrode, membrane, nicotinamide, phosphate sensor, environmental analysis

## Abstract

Phosphorus is not only an import nutrient to aquatic habitats, but it also acts as a growth inhibitor in aquatic ecosystems; however, it also aggravates environmental issues, such as eutrophication. There is a growing interest in rapid phosphorus detection to manage and protect water resources. Due to the large molecular structure and high hydration energy of phosphate ions, ion-selective electrodes (ISEs) remain in their infancy for real-time measurements in terms of practical application. In this study, a newly developed ionophore based on a biomimetic nicotinamide functional group was used to detect phosphate selectively, displaying efficient binding through charge interactions and hydrogen bonds. The ISE membrane containing silicone rubber demonstrated an effective detection performance over a long period of time. With a dynamic range between 10^−6^ and 10^−2^ M and a limit of detection of 0.85 × 10^−6^ M (26 μg/L), the newly synthesized ISE membranes demonstrated selectivity for phosphate ions over other ions, including acetate, sulfate, and chloride.

## 1. Introduction

Growth-limiting nutrients of aquatic habitats, such as carbon, nitrogen, and phosphorus, play important roles in aquatic ecosystems. An accurate assessment of growth-limiting nutrients can provide a better understanding of aquatic ecosystems. It is crucial to accurately measure phosphorus chemically and biochemically. Phosphorus often causes eutrophication owing to its excessive loading into water bodies, which impairs the aquatic environment [1,2]. Environmental agencies in many countries have established recommended limits for total phosphorus or phosphate concentrations and standard protocols for determining their concentrations to manage and protect water resources [3,4,5]. However, these standard protocols mostly require sample collection, reaction with redox agents or coloring agents under harsh conditions in the laboratory, and spectroscopic measurements within a certain time period [6,7].Therefore, this method is not suitable for the effective real-time monitoring of phosphate ion concentration changes because of its complex and time-consuming steps. A number of electrochemical and spectrophotometric commercial sensors offer the seamless monitoring of nutrients such as NH_4_^+^, K^+^, Na^+^, Mg^2+^, Ca^2+^, Cl^−^, NO_3_^−^, CN^−^, and F^−^ [8,9,10,11,12,13,14]. However, currently, no commercial phosphate sensors that can handle real-time analysis exist in the field.

Phosphorus is mostly present in the form of phosphate ions, which are considered to be relatively large ions similar to sulfate, acetate, and perchlorate ions. Phosphorous’s large hydration energy renders it difficult to develop a sensor based on hydrogen bonding between the sensing material and the analyte [15,16]. Despite these challenges, researchers have been developing phosphate sensors based on spectrophotometric sensors using colorimetric or photoluminescent probes and electrochemical sensors, such as potentiometric or voltametric sensors [17,18,19,20,21,22,23,24]. Spectrophotometric sensors have a relatively low limit of detection (LOD) but a narrow linear concentration range with respect to their sensor performance. However, electrochemical sensors have a wide linear relationship between phosphate concentrations and sensor output signals. Pioneering research on phosphate sensors based on electrochemical principles has focused on ion-selective electrode (ISE) sensors using urea or thiourea-functionalized calix[4]arene as an ionophore [6,25]. The ionophore possesses well-organized interaction sites for spherical or tetragonally structured ions through hydrogen bonds [26]. The ISE sensor using calix[4]arene shows an ideal Nernst behavior in the 10^−5^– 10^−1^ M concentration range and an LOD of 5 × 10^−5^ M for its phosphate concentration [6]. However, its inferior sensitivity to phosphate, rather than perchlorate, remains to be improved.

In this study, ionophores with nicotinamide functional groups were designed and synthesized to develop an ISE sensor that detects phosphate effectively, and their phosphate detection characteristics were also studied. As an important biomaterial, nicotinamide is a water-soluble form of vitamin used as a precursor of the nicotinamide-adenine dinucleotide. Specific hydrogen bonding between nicotinamide and the phosphates of lipids plays a critical role as nicotinamide effectively penetrates phospholipids in biological systems [27,28]. This study demonstrates an ISE sensor consisting of a nicotinamide-based ionophore that detects phosphate selectively and sensitively by imitating the specific interaction in a biological system and examines its characteristics. Furthermore, the long-term performance stability of the sensor was achieved by optimizing the ISE membrane constituents.

## 2. Materials and Methods

Poly-vinyl chloride (PVC), 2-nitrophenyl octyl ether (NPOE), tetradodecylammonium bromide (TDAB), potassium chloride, tetrahydrofuran (THF), dimethyl sulfoxide (DMSO), acetonitrile (ACN), ethanol (EtOH), and potassium phosphate monobasic were purchased from Sigma Aldrich (Merck Korea, Seoul, Korea). Silicone rubber RTV 3140 was obtained from DOWSIL (Dow, MI, USA). Nicotinamide, 1,3-bis (bromomethyl) benzene and potassium hexafluorophosphate (KPF_6_) obtained from Tokyo Chemical Industry CO., Ltd (SEJIN CI Co., Ltd., Seoul, Korea). All aqueous solutions were prepared using deionized water (resistivity of 18.2 M Ω cm). All chemicals were used without further purification.

### 2.1. Synthesis of Ionophores

**Scheme 1:**
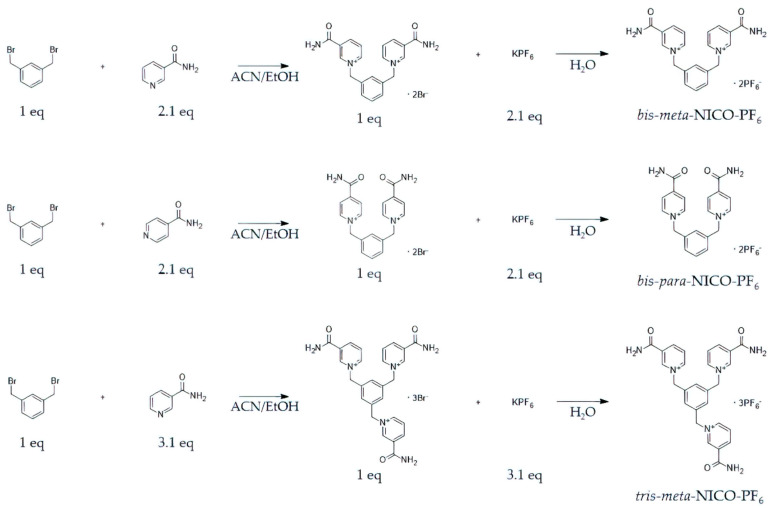
Synthetic scheme of phosphate ionophores with nicotinamide functional groups. The symbol “eq” stands for equivalent weight.

*1,3-Phenylenebis (methylene) [3-(N,N-diethyl)carbamoylpyridinium] bromide (bis-meta-*NICO-Br) was prepared via a method used in previous study [29]. Nicotinamide (2.00 g, 16.7 mmol) was added to a solution of 1,3-bis (bromomethyl)benzene (2.00 g, 7.06 mmol) in a solvent mixture of ACN (15 mL) and EtOH (35 mL) (Scheme 1). The reaction mixture was stirred for 3 h at 82 °C and then cooled to room temperature. The white precipitates were filtered and washed thrice with cold ethanol and then dried in an oven at 60 °C (1.90 g, yield: 49%). ^1^H nuclear magnetic resonance (NMR, 300 MHz, DMSO-*d*_6_): δ 9.64 (s, 2H, -NCH-), δ 9.32 (d, 2H, -CH-), δ 9.01 (d, 2H, -CH-), δ 8.62 (s, 2H, -NH_2_), δ 8.30 (t, 2H, -CH-), δ 8.20 (s, 2H -NH_2_), δ 7.79 (s, 1H, Ar-H), δ 7.60 (d, 2H, Ar-H), δ 7.53 (t, 1H, Ar-H), and δ 5.96 (s, 4H, -CH_2_-).

*Synthesis of bis-meta-*NICO-PF_6_: The counter anions of *bis-meta-*NICO-Br, which is a cation, were exchanged with hexafluorophosphate. This additional procedure was conducted to improve the solubility of the ionophore in common organic solvents and prepare a homogeneous ISE membrane (Scheme 1). An aqueous solution (20 mL) of *bis-meta-*NICO-Br (0.500 g, 3.20 mmol) was added to an aqueous solution (30 mL) of KPF_6_ (2.55 g, 13.7 mmol). The reaction mixture was then stirred for 2 h at room temperature (Scheme 1). The white precipitates were filtered, washed thrice, and dried in an oven at 60 °C (0.466 g, yield: 73%). ^1^H NMR (300 MHz, DMSO-*d*_6_): δ 9.55 (s, 2H, -NCH-), δ 9.22 (d, 2H, -CH-), δ 8.97 (d, 2H, -CH-), δ 8.57 (s, 2H, -NH_2_), δ 8.30 (t, 2H, -CH-), δ 8.20 (s, 2H -NH_2_), δ 7.67 (s, 1H, Ar-H), δ 7.56 (d, 2H, Ar-H), δ 7.54 (t, 1H, Ar-H), and δ 5.92 (s, 4H, -CH_2_-). MALDI-TOF mass (m/z) calculated—493.3642; observed—492.6193 [M^+^PF_6_^−^] (Figure S1).

*Bis-para-*NICO isomer was prepared by following the same procedure as that of *bis-meta-*NICO using *p*-nicotinamide as a starting material (see Supporting Information). The synthesis of *tris-meta-*NICO-PF_6_ was performed by switching 1,3-bis (bromomethyl) benzene with 1,3,5-tris (bromomethyl)benzene (Scheme 1).

### 2.2. Membrane

**Figure 1:**
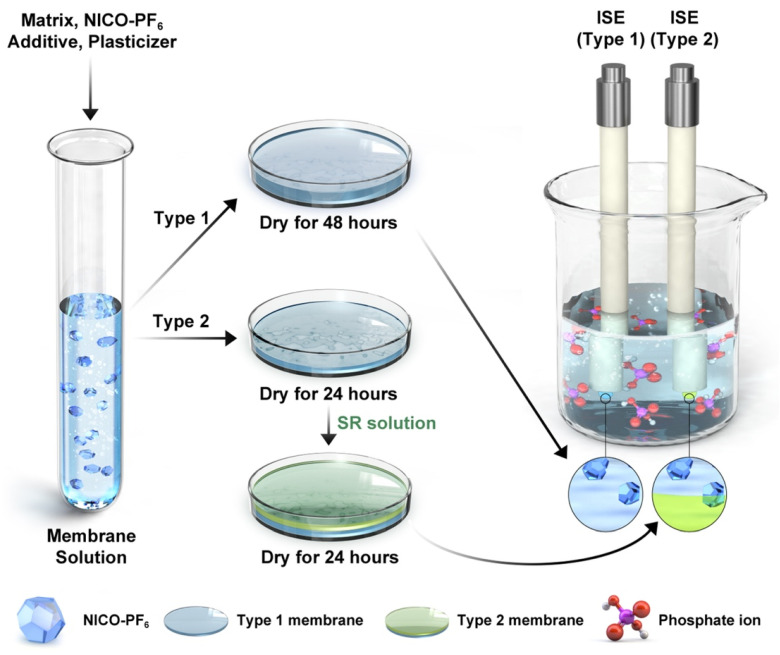
Fabrication of Type 1 and Type 2 ISE membranes composed of ionophore (*bis-meta-*NICO-PF_6_).

To fabricate the Type 1 ISE membrane, we first prepared a PVC solution by dissolving PVC (33 mg) in THF (400 μL) and then vortexing it for 2 h. Thereafter, 1 mg of *bis-meta-*NICO-PF_6_ was dissolved in THF (100 μL) and DMSO (40 μL) to obtain *bis-meta-*NICO-PF_6_ solution, which was further mixed with the PVC solution and stirred for 1 h. TDAB (1 mg), as an additive in THF (100 μL), was mixed with PVC/*bis-meta-*NICO-PF_6_ solution and stirred for 1 h. Furthermore, NPOE (1.04 g/mL, 66 μL) was added to the PVC/*bis-meta-*NICO-PF_6_/additive solution and stirred for 2 h. The resulting solution (membrane solution) was dried at room temperature for 48 h using a glass ring of a predetermined size [30]. A further layer of silicone rubber (SR) solution was prepared for developing Type 2 membrane (Figure 1). The SR solution was prepared by mixing the membrane solution and RTV 3140 (50 mg/mL, dissolved in THF) at a 1:1 volume ratio [31]. To prepare the Type 2 ISE membrane, the membrane solution (360 μL) was dried at room temperature for 24 h and then covered with the SR solution (240 μL), followed by drying for an additional 24 h. When the Type 2 ISE membrane was evaluated, the layer formed by drying the SR solution was exposed to the analyte solution.

### 2.3. Characterization

The NMR data—including 2D experiments and NMR titration studies for the compounds *bis-meta*-NICO-PF_6_, *tris-meta*-NICO-PF_6_, and *bis-para*-NICO-PF_6_—were collected in samples dissolved in DMSO-*d*_6_ with ^1^H referencing to the residual solvent peak at 2.50 ppm on an NMR spectrometer (Fourier 300, Bruker, Billerica, MA, USA). The following abbreviations were used to indicate the multiplicity in the NMR spectra: s = singlet, d = doublet, and t = triplet signal. The structure of the membrane and its binding behavior to the ions were verified using Fourier transform infrared (FT-IR) spectroscopy (ALPHA-P, USA).

### 2.4. Electromotive Force (EMF) Measurement

The EMF was evaluated to quantify the strength and selectivity of the ion interactions in a liquid membrane. A potentiometer (KST101A, Kosentech, Korea) with a computer-based data-gathering system was used to examine the EMFs of each sample. The measurement setup featured a double-junction Ag/AgCl electrode saturated with KCl as a reference electrode from Thermo Fisher Scientific. The EMF of the stabilized ISEs was measured after 5-min incubation in deionized water. The EMF or membrane potential (*E*) was calculated for sample concentrations (*C*) ranging from 10^−6^ M to 10^−2^ M as follows:

(1) $$E=E^{0}+\frac{RT}{zF}logC=E_{0}+S\times logC$$

where *R* is the gas constant (8.314 J/K). mol), *T* is the temperature (K), *F* is the Faraday constant (96,500 C/mol), and z is the charge of the analyte ion. With the exception of the membrane–sample solution interface, the potential differences of all other interfaces are added together to obtain the constant term *E*^0^, which is unique to the analyte [32]. A plot of *E* versus logC demonstrates that the measured *E* is proportional to the logarithm of the analyte concentration with a slope (S).

The ISE capacity to match the principal ions in a mixed solution is measured by its selectivity coefficient ($k_{ij}^{pot}$) [33,34]. The selectivity coefficient was obtained using the separation method at the same concentrations ranging between 10^−6^ and 10^−2^ M. *E*_1_ and *E*_2_ are the measured membrane potentials for a solution containing the salt of the primary ion *i* and interfering ion *j,* respectively; $z_{i}$ is the charge of the primary ion; S is the slope of the linear part of the electrode calibration curve; and ΔE is the potential difference between ions *j* and *i.*

(2) $$\log k_{ij}^{pot}=\frac{\left(E_{2}-E_{1}\right)z_{i}F}{2.303RT}=\frac{\Delta E}{S}$$

## 3. Results

### 3.1. Synthesis of Ionophore and Its Interaction with Phosphate

**Figure 2:**
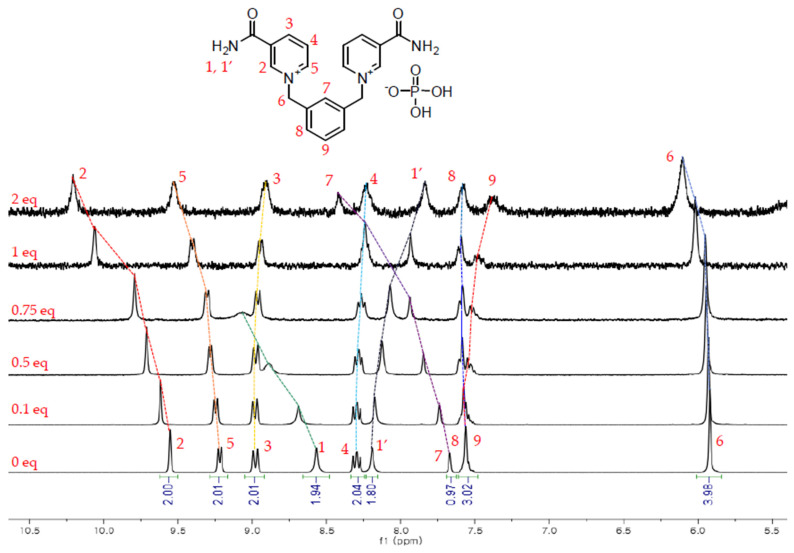
^1^H-NMR spectra of bis-meta-NICO-PF_6_ during phosphate titration.

The phosphate receptors were designed to have cationic moieties, such as pyridinium and imidazolium. In the preliminary phosphate titration tests, nicotinamide-type receptors showed a high performance compared with the other ionophores owing to the additional H-bonding sites. Significantly, the bis-nicotinamide-based ionophore (*bis-meta*-NICO-PF_6_, Figure 2) exhibited stronger interactions with phosphate ions than *tris-meta*-NICO-PF_6_ (Figure S2). The different positions of the amide groups, the meta- and para-isomers, were also compared through phosphate titration studies (Figure S3). Based on the findings of a preliminary NMR titration investigation, *bis-meta*-NICO-PF_6_ was chosen as the best molecular design among the ionophores in this study for the adhesion of an ionophore to a phosphate ion. The optimized modeling structure of the *bis-meta*-NICO-PF_6_ was obtained by repeating the energy minimization process five times on Chem3D software (Figure S4). In a three-dimensional modeling structure, the two arms of the receptor can be aligned to form a binding pocket, which is flexible and fits well with a phosphate ion. The groove of the ionophore provides cationic charges for electrostatic attraction and hydrogen bonding. The protons on the ionophore molecule were determined based on the two-dimensional-NMR interpretation as shown in Figures S5 and S6. In phosphate titration studies, 2-H, which is adjacent to the amide group and positively charged nitrogen, was significantly affected by the phosphate ion, and large downfield shifts were observed (Figure 2). Similarly, 5-H also was significantly shifted downfield. However, 3-H and 4-H showed substantially smaller upfield shifts as they were separated from the amide group. This tendency indicates that charge interactions and hydrogen bonding with amide groups play an important role in facilitating the interaction between the ionophore and phosphate ions. Significantly, two amide protons (1-H and 1-H’) split each other, and a 1-H proton peak initially appeared at 8.6 ppm and gradually moved downfield and broadened to 9.1 ppm when a 0.75 equivalent of phosphate was added. The other amide proton, 1-H’, moved in the opposite direction. A fast and dynamic exchange processes may be involved, and the large chemical-shift indicates a strong interaction between amide groups and phosphate anions. The 7-H showed the largest downfield shift, which strongly supports the formation of a binding pocket around 2-H, 5-H, and 7-H by a phosphate anion.

Phosphate titration investigations with a variety of anions, including CH_3_COO^−^, NO_3_^−^, SO_4_^2−^, Br^−^, and Cl^−^, were conducted (Figures S7–S11). The results of the NMR titration experiments are summarized and listed as chemical shift changes (Figure S12). The chemical shifts of all the protons in *bis-meta*-NICO-PF_6_ were investigated when 1 eq of an anion was added. Large downfield shifts and their wide distribution were observed for 2-H, 5-H, and 7-H in all cases, owing to the strong interaction between *bis-meta*-NICO-PF_6_ and an anion. Figure S13 shows plots only for 7-H to compare and estimate the relative binding affinity upon increasing the amount of guest anions. These results are very useful for determining the selectivity order for anions in solution, which is determined as H_2_PO_4_^−^ > Cl^−^ > SO_4_^2−^ > CH_3_COO^−^ > NO_3_^−^ > Br^−^.

### 3.2. Membrane Characteristics

**Figure 3:**
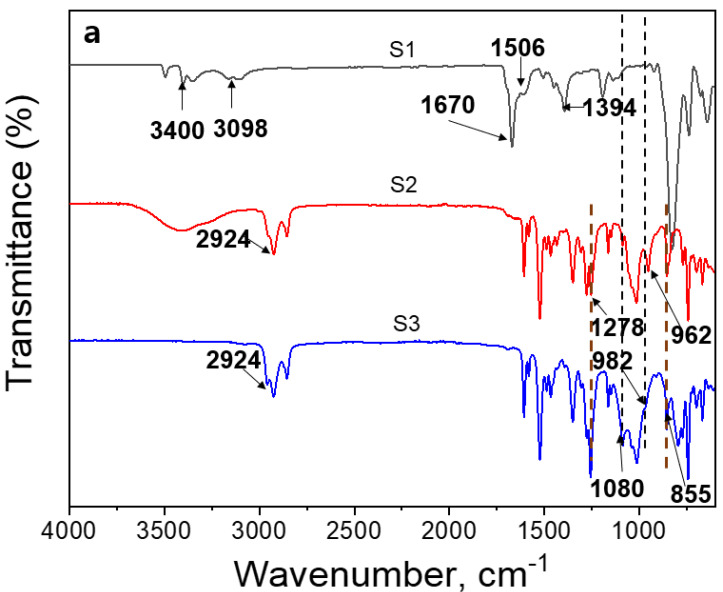
FT-IR spectra of the *bis-meta-*NICO-PF_6_ (S1) and Type 2 membrane before (S2) and after incubation (S3) with phosphate.

FT-IR spectroscopic analyses were performed to verify the interactions between *bis-meta*-NICO-PF_6_ and phosphate ions in the Type 2 membrane. Figure 3 shows the FT-IR spectra of the *bis-meta-*NICO-PF_6_ (S1), Type 2 membrane (S2), and Type 2 membrane reacted with 0.5 M phosphate ions (S3). The peaks corresponding to nicotinamide were clearly observed in S1 at 3400 (N–H), 1670 (C=O), 1506 (C=C), and 1394 cm^−1^ (C–N) [35]. The spectra (S2, S3) of the Type 2 membranes in Figure 3 showed several vibration bands from PVC and silicone rubber: 2924 (C–H stretching), 1278 (C–H rocking), 962 (C–H wagging), 855 (C–Cl), and ca. 1000 cm^−1^ (Si–O–Si) [36,37]. Additionally, it is known that bands for P–O from phosphate ions can be seen at approximately 1070 and 982 cm^−1^ [38]. The peaks in S3 at 1080 and 982 cm^−1^ can be attributed to the presence of phosphate in the Type 2 membrane.

### 3.3. Evaluation of ISE Response

**Figure 4:**
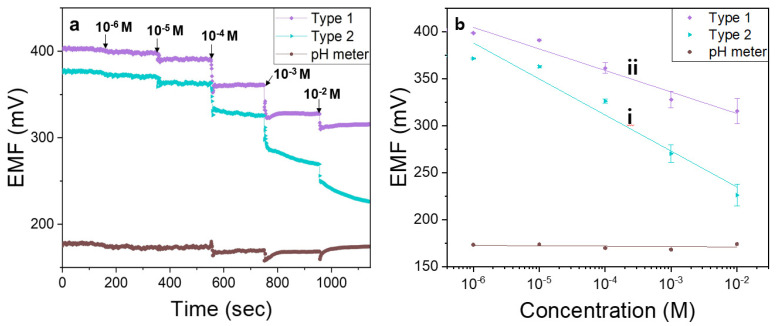
Calibration curve of two types of ISEs: (**a**) EMF response from 10^−6^ to 10^−2^ M KH_2_PO_4_ and (**b**) plot of EMF versus logarithmic concentration.

The EMF responses were measured before and after the interaction between ISE and H_2_PO_4_ at various concentrations ranging between 10^−6^ and 10^−2^ M. The EMF values were obtained by measuring the differences between the internal and external potentials of the membrane during the interaction between H_2_PO_4_ and *bis-meta-*NICO-PF_6_. Figure 4a shows that the pH sensor barely changed in response to the phosphate concentration; however, the EMF values linearly decreased with an increasing H_2_PO_4_ concentration. The EMF was plotted against the logarithm of the H_2_PO_4_ concentration (Figure 4b and Figure S14). Therefore, we can deduce that the change in the signal was caused by a change in the amount of phosphate rather than a change in the hydrogen ions. The linear dependence between the EMF and H_2_PO_4_ concentration led to a regression equation of y = −22.9 logx + 0.3 with a correlation coefficient of 0.96 for Type 1. With a correlation coefficient of 0.95, the regression equation y = −38.3 logx + 158.2 was obtained for Type 2.

The reproducibility, expressed in terms of the relative standard deviation, was approximately 2.9% (n = 3) at a H_2_PO_4_ concentration of 5.0 μM. The LOD value of phosphate for the Type 2 membrane was 0.85 × 10^−6^ M (26 μg/L) (Figure 4b(i)), using the formula LOD = 3 σ/k, where *σ* is the standard deviation and *k* is the slope [39]. However, the phosphate detection without an SR layer membrane (Type 1) demonstrated that the linear range was between 10^−6^ and 10^−2^ M, and the LOD was approximately 1.54 × 10^−6^ M (47 μg/L) (Figure 4b(ii)). The Type 2 membrane achieved an LOD three times lower than that of the Type 1 membrane, demonstrating that the SR layer effectively prevented the leakage of *bis-meta-*NICO-PF_6_ and maintained the potential difference.

**Figure 5:**
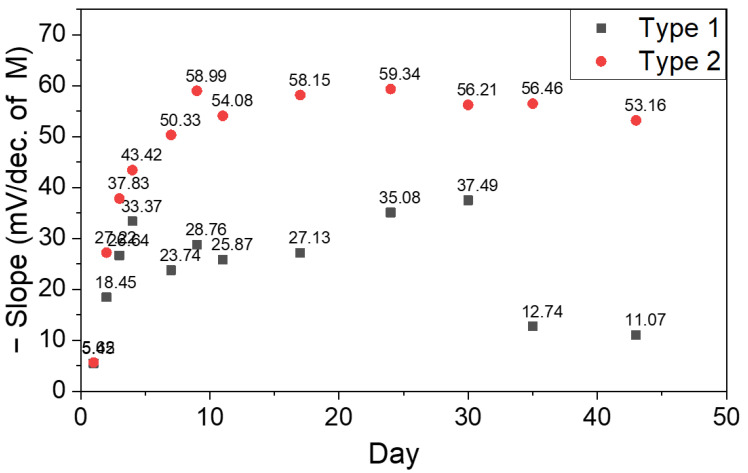
Durability measurements taken over a period of 43 days for Type 1 and Type 2 ISEs.

During a long period of measurements, the phosphate ion-sensing behaviors of the Type 1 and Type 2 membranes were different. When the measurements were taken continuously over a period of five days, the Type 2 membrane exhibited a larger slope than the Type 1 membrane because of its higher sensitivity to the changes in the phosphate concentration. Figure S15 shows that the results from Type 1 and Type 2 had statistically significant differences according to the *p*-value obtained under the equal variance condition of the independent sample T test, which was 0.000761. The slope of Equation (1) was evaluated over a period of more than 40 days to assess the long-term durability of the Type 1 and 2 membranes (Figure 5). The slopes exhibited a gradual increase for approximately 5 to 10 days, allowing the membrane matrix’s aging or component reorganization to occur. The membrane matrix demonstrated a phenomenon of gradual sensitivity enhancement based on the rising slope, as opposed to most ISE matrixes, which showed a performance loss with aging [11]. After 30 days, the Type 1 slope was reduced by 70% (from −37.49 to −11.07), but the Type 2 slope was only diminished by 5% (−56.21 to −53.16). According to previous studies, the good sensitivity and durability of the Type 2 membrane can be attributed to the ability of the SR layer to prevent *bis-meta-*NICO-PF_6_ leakage into the matrix [31].

The selectivity coefficient was determined to verify the binding constants of various anions. A separate solution method was used to calculate the coefficient [33,34]. The Type 1 membrane showed higher affinities for NO_3_^−^ and Cl^−^ than H_2_PO_4_^−^, but the selectivity coefficient of phosphate to both NO_3_^−^ and Cl^−^ had improved in Type 2. However, NO_3_^−^ acted as an interfering ion for H_2_PO_4_^−^ detection in both Type 1 and Type 2 [40], which necessitates a more scrutinized approach, such as molecular imprinting. The selectivity coefficient for the Type 2 ISE changed in the following order: H_2_PO_4_^−^ ≥ NO_3_^−^ > Cl^−^ > SO_4_^2−^ > CH_3_COO^−^. Furthermore, NO_3_^−^ had the second-lowest affinity for NICO-PF_6_ in the solution-based NMR titration experiments. However, NO_3_^−^ demonstrated a similar attraction to NICO-PF_6_ in the Type 2 membrane. These results may be attributed to the differences between the solution and liquid membrane states. Further investigations should be conducted on the interactions between NO_3_^−^ and other components of the Type 2 membranes, such as PVC, additives, plasticizers, and RTV 3140 as SR.

Table 1 presents a comparison of the analytical properties of the Type 1 and Type 2 ISE and other H_2_PO_4_−ISEs, demonstrating that they have equivalent linear ranges, slopes, and selectivity values ($\log k_{ij}^{pot}$) [40,41,42,43]. Furthermore, Type 2 outperformed the other sensors in terms of stability and LOD. Therefore, the newly synthesized ionophores inspired by the nicotinamide moiety owing to their multiple binding modes through hydrogen bonds and charge interactions, as well as effective matrix components such as SR that prevent ionophores from dissolving, are considered effective for their unique characteristics.

## 4. Conclusions

The specific affinity between nicotinamide and phosphate through hydrogen bonding and charge interactions was utilized to develop an effective ionophore that can be applied in an ISE-based phosphate sensor. Several analogs of ionophores, including *bis*-, *tris*-, *meta*-, and *para*-NICO, which include the nicotinamide functional group, were synthesized and their affinities to phosphate in the solution phase were investigated using NMR spectroscopic analyses. In this study, the most efficient ionophore was *bis-meta-*NICO-PF_6_, which had a stronger affinity for phosphate than Cl^−^, SO_4_^2−^, CH_3_COO^−^, NO_3_^−^, and Br^−^ in the solution phase. *Bis-meta-*NICO-PF_6_ had to be embedded into the membrane using carefully formulated membrane components, such as PVC, NPOE as a plasticizer, and DTAB as an additive; its affinity for phosphate ions in the membrane was practically comparable with that of the ionophore in the solution phase with the only exception being NO_3_^-^. Further studies should be conducted to understand the different affinity behaviors of *bis-meta-*NICO-PF_6_ for NO_3_^−^ in solution and in the ISE membrane. In addition, an SR layer was introduced to prevent the elution of *bis-meta-*NICO-PF_6_ from the developed membrane and was found to maintain the sensor performance for a long time-period, namely, over 40 days. Therefore, we developed a Type 2 membrane based on ISE with an LOD value of 0.85 × 10^−6^ M for phosphate, which is one of the best LOD values reported thus far for phosphate sensors in the field of ISEs. With its selectivity, sensitivity, and durability characteristics, the newly synthesized ionophore, *bis-meta-*NICO-PF_6_, can be used for the seamless and real-time monitoring of phosphate concentration changes in the field.

## Figures and Tables

**Table 1:** Comparison of ISE characteristics of Type 1 and 2 with other research results.

	Linear Range(M)	Slope (mV/decade)	LOD (μM)	Stability(Day)	Selectivity					
					CH_3_ COO^−^	Cl^−^	NO_3_^−^	Br^−^	SO_4_^2−^	HCO_3_^−^
ref [40]	10^−6^–10^−1^	−59.0	1.6	4.8	-	−2.9	−2.1	−2.5	−3.9	-
ref [41]	10^−6^–10^−1^	−55.7	2.14	-	-	−2.37	-	-	−2.52	−2.31
ref [42]	10^−5^–10^−1^	−37.2	10	20	-	1.82	−2.10	2.04	-	−1.67
ref [43]	10^−4^–10^−1^	−60.0	70	-	-	−1.5	−2.5	−1.4	−2.3	−0.3
Type 1 *	10^−6^–10^−2^	−23.5	1.54	30	−6.09	0.02	1.71	-	−0.68	-
Type 2 *	10^−6^–10^−2^	−53.3	0.85	40	−16.3	−1.32	−0.06	-	−2.23	-

* This study.

## Data Availability

Not applicable.

## Supplementary Materials

The following supporting information can be downloaded at: https://www.mdpi.com/article/10.3390/polym14163392/s1, Figure S1. (a) ^1^H-NMR spectrum of *bis-meta*-NICO-PF_6_ ionophore. (b) MALDI-TOF spectrum of *bis-meta-*NICO-PF_6_ m/z=492.6193 [M^+^PF_6_^−^].; Figure S2. ^1^H-NMR spectra of *tris-meta*-NICO-PF_6_ under phosphate titration.; Figure S3. ^1^H-NMR spectra of *bis-para*-NICO-PF_6_ under phosphate titration study.; Figure S4. Three-dimensional modeling of *bis-meta*-NICO-PF_6_ binding phosphate.; Figure S5. 2-D NOESY NMR spectrum of *bis-meta*-NICO-PF_6_.; Figure S6. 2-D NOESY NMR spectrum of *bis-meta*-NICO-PF_6_ with phosphate ion.; Figure S7. Acetate titration to *bis-meta*-NICO-PF_6_ in DMSO-*d*_6_.; Figure S8. Nitrate titration to *bis-meta*-NICO-PF_6_ in DMSO-*d*_6_.; Figure S9. Sulfate titration to *bis-meta*-NICO-PF_6_ in DMSO-*d*_6_.; Figure S10. Bromide titration to *bis-meta*-NICO-PF_6_ in DMSO-*d*_6_.; Figure S11. Chloride titration to *bis-meta*-NICO-PF_6_ in DMSO-*d*_6_.; Figure S12. Chemical shift change distribution of *bis-meta*-NICO-PF_6_ upon 1 equivalent of anion addition.; Figure S13. Chemical shift change profile of 7-H on *bis-meta*-NICO-PF_6_ upon anion titration.; Figure S14. Type 1 and Type 2 ISE reproducibility across production batches.; Figure S15. Slope comparison of Type 1 and Type 2 during five consecutive days.
