# Supplementary material for: Ion-Selective Electrode Based on a Novel Biomimetic Nicotinamide Compound for Phosphate Ion Sensor
Source: Polymers (Basel). 2022 Aug 19;14(16):3392. doi: 10.3390/polym14163392 (PMC9414682; doi:10.3390/polym14163392)
Supplement: Supplementary file 1 [file polymers-14-03392-s001.zip › polymers-1856739-supplementary.pdf]

Supporting Information

Ion-Selective Electrode Based on a Novel Biomimetic Nicotinamide Compound for Phosphate Ion Sensor

Bongjin Jeong [1,†], Jin Seong Oh [2,†] , Do Yeob Kim [1], , Dong Gyu Kim [2], Young Il Kim [2], Jungseok Heo [2,*] and Hyung-Kun Lee [1,*]

[1] ICT Creative Research Laboratory, Electronics & Telecommunications Research Institute, Daejeon 34129, Korea
[2] Department of Chemistry, Chungnam National University, Daejeon 34134, Korea
* Correspondence: jungseokheo@cnu.ac.kr (J.H.); hklee@etri.re.kr (H.-K.L.)
† These authors contributed equally to this work.

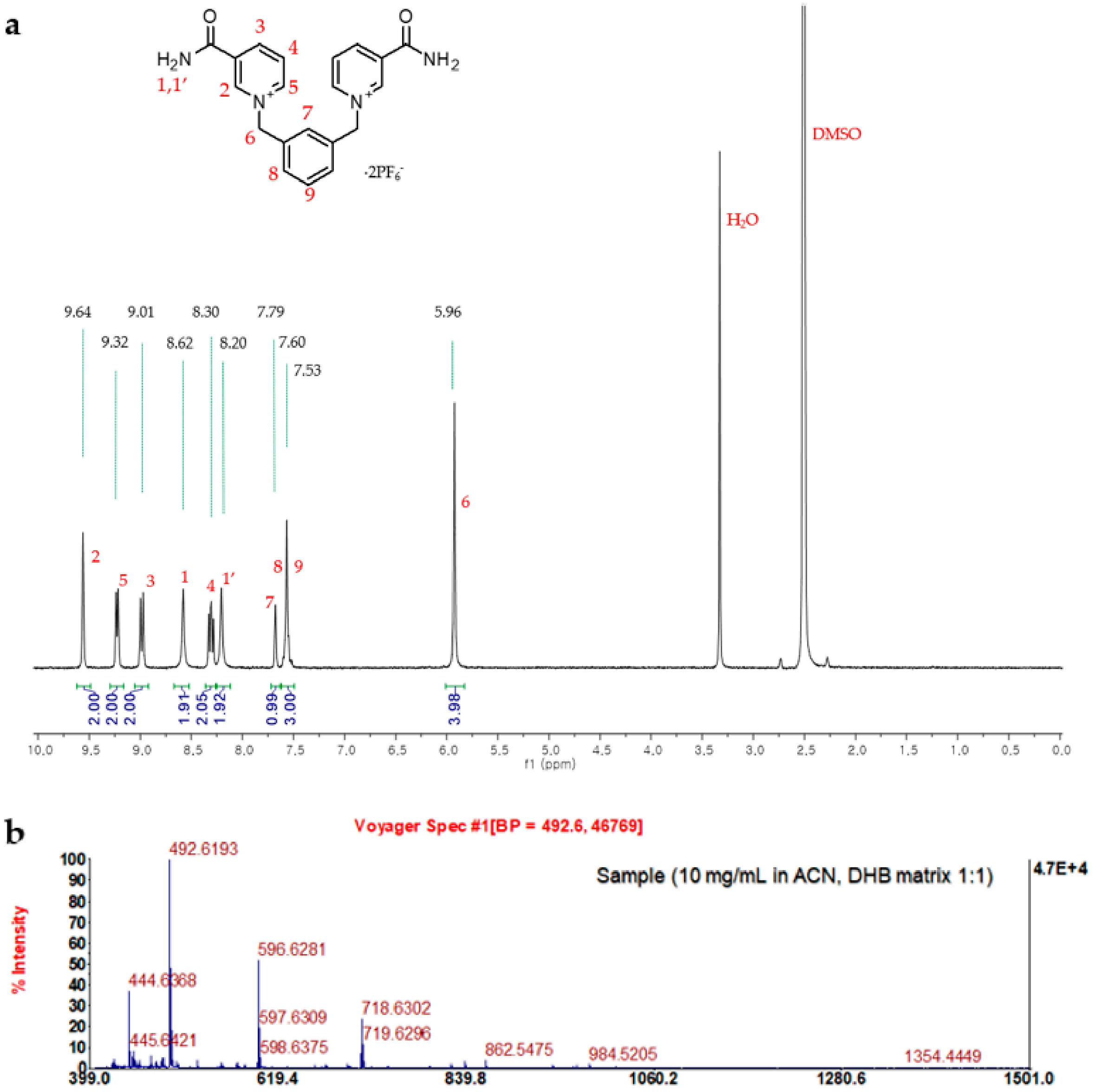

Figure S1. (a) ^{1}H-NMR spectrum of *bis-meta*-NICO-PF_6 ionophore. (b) MALDI-TOF spectrum of *bis-meta*-NICO-PF_6 m/z=492.6193 [$M^+PF_6^-$].

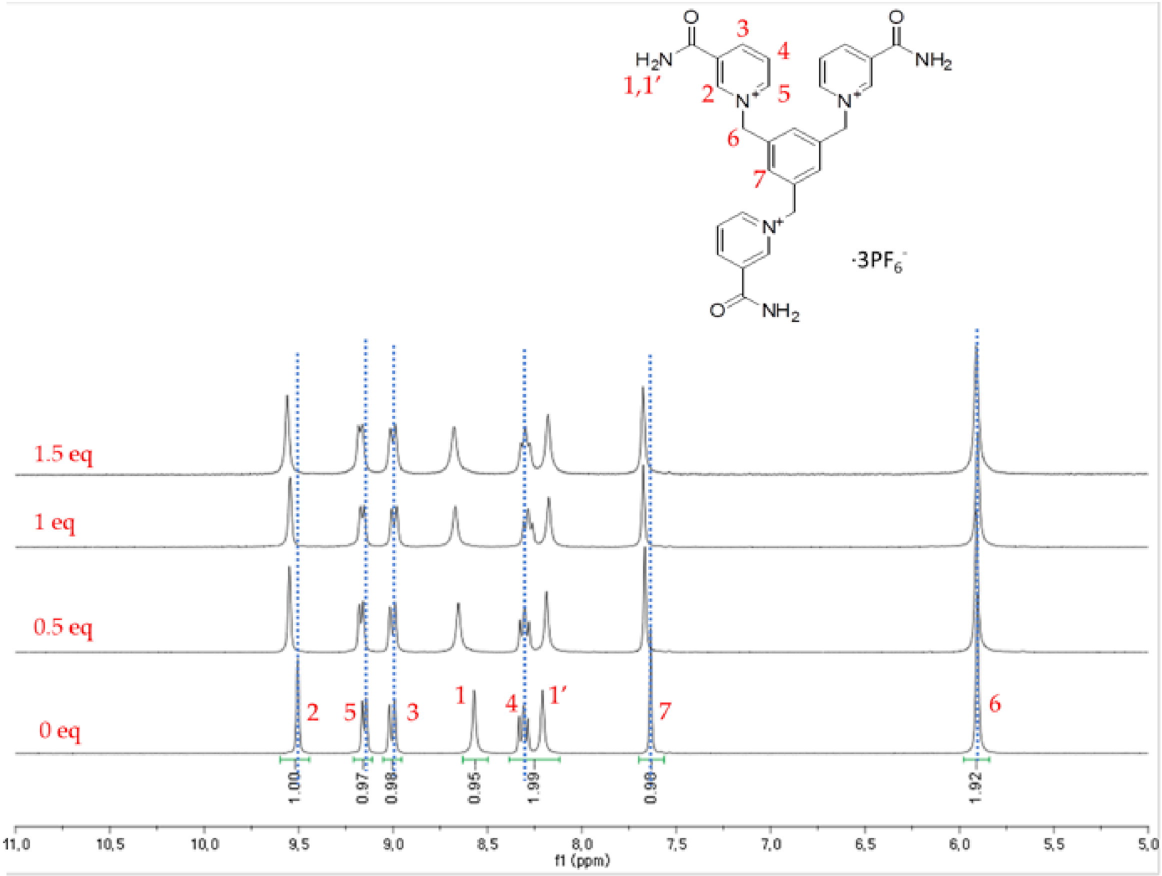

Figure S2. ^{1}H-NMR spectra of *tris-meta*-NICO-PF_6 under phosphate titration.

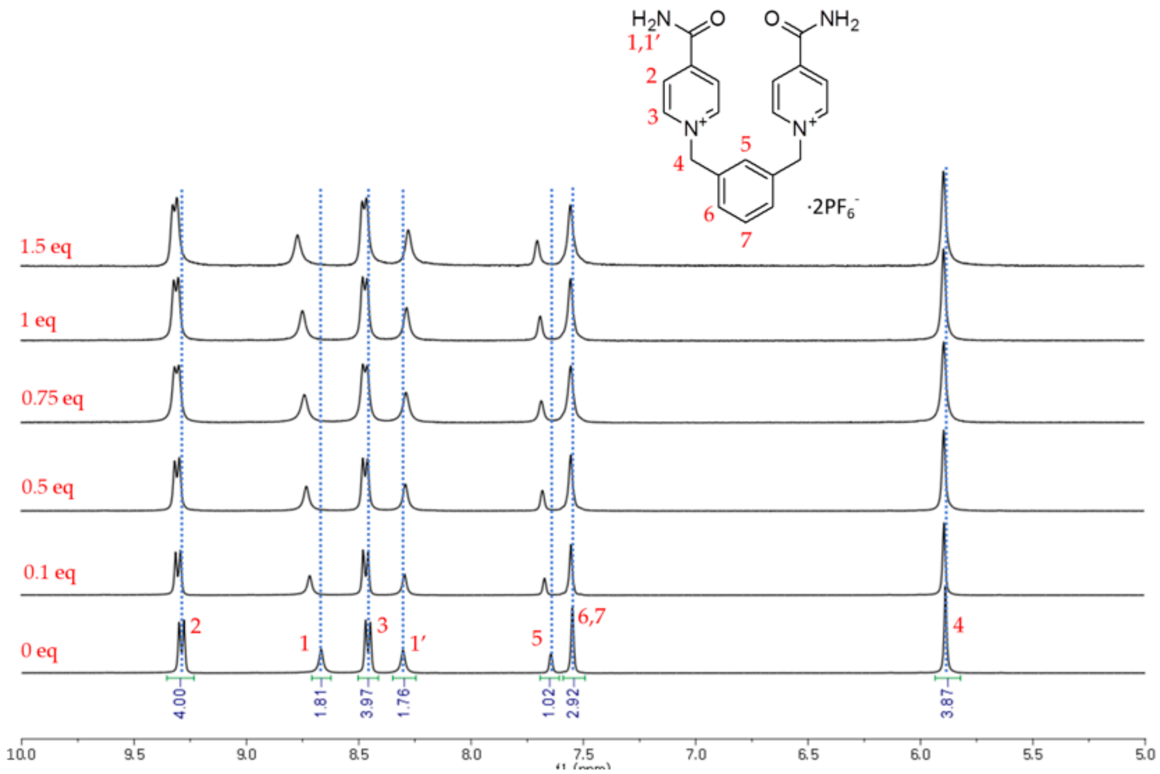

Figure S3. ^{1}H-NMR spectra of *bis-para*-NICO-PF6 under phosphate titration study.

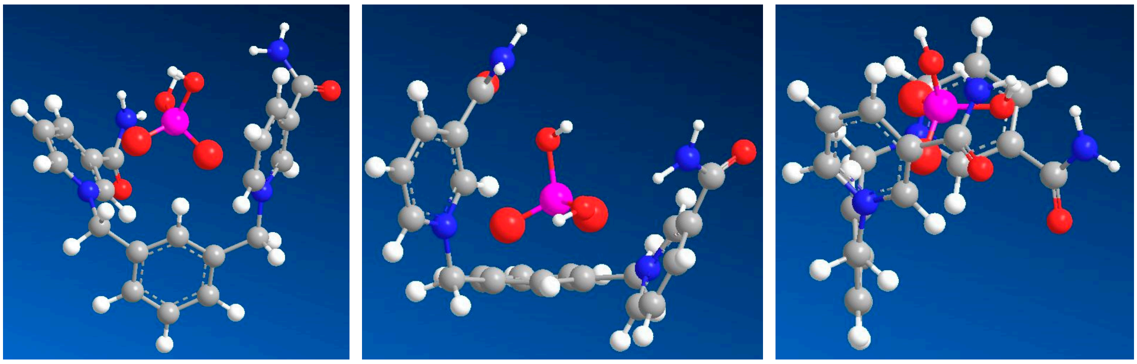

Figure S4. Three-dimensional modeling of *bis-meta*-NICO-PF6 binding phosphate.

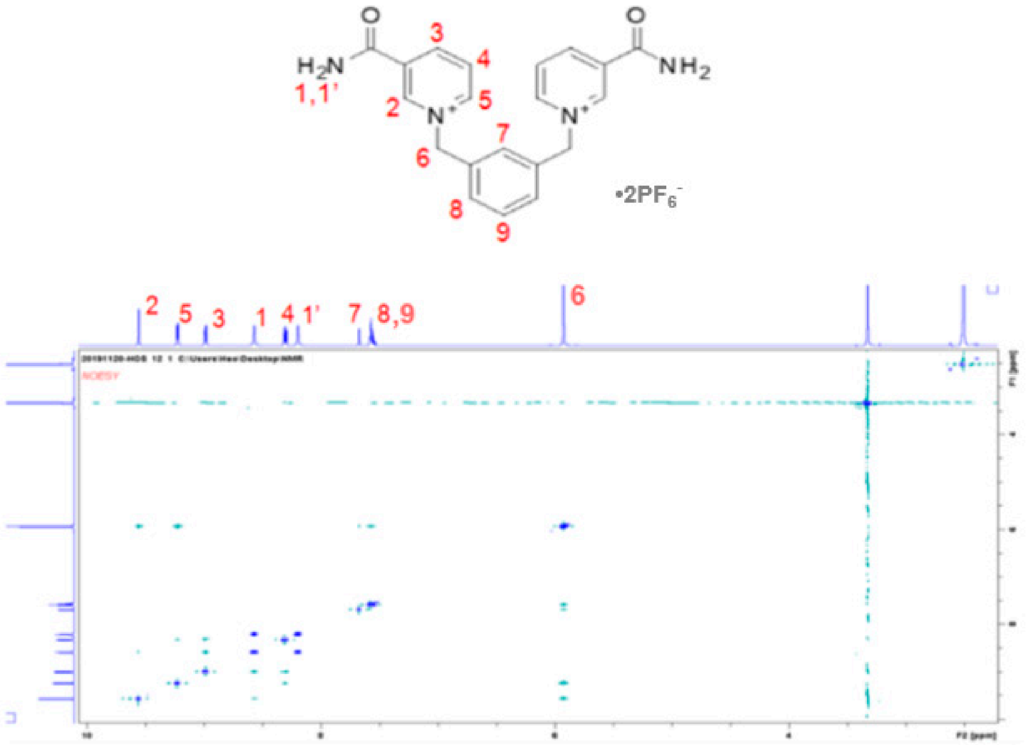

Figure S5. 2-D NOESY NMR spectrum of *bis-meta*-NICO-PF_6 .

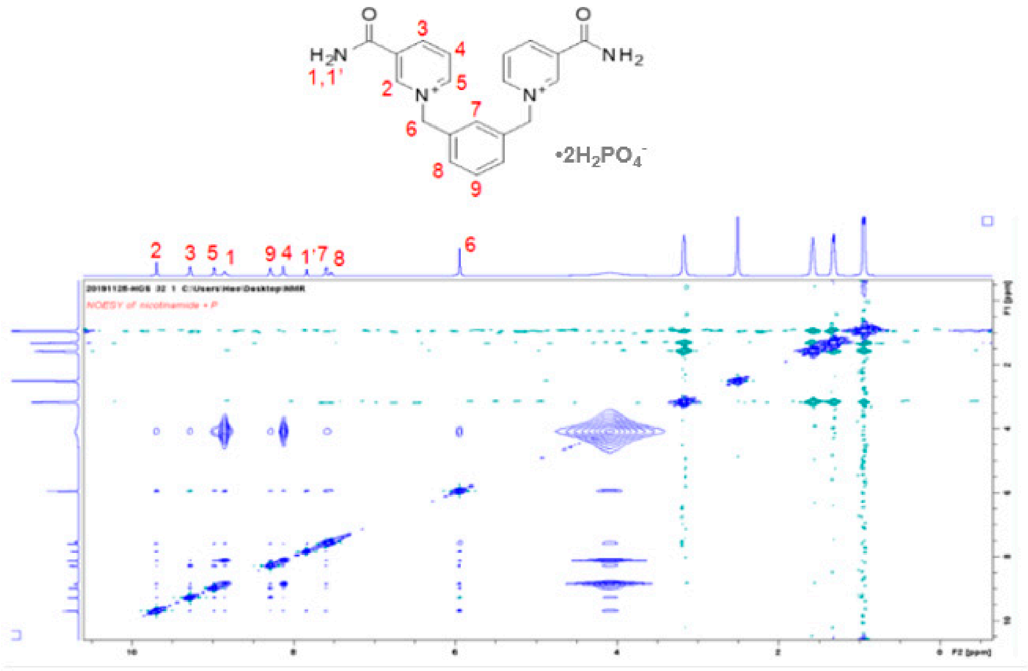

Figure S6. 2-D NOESY NMR spectrum of *bis-meta*-NICO-PF_6 with phosphate ion.

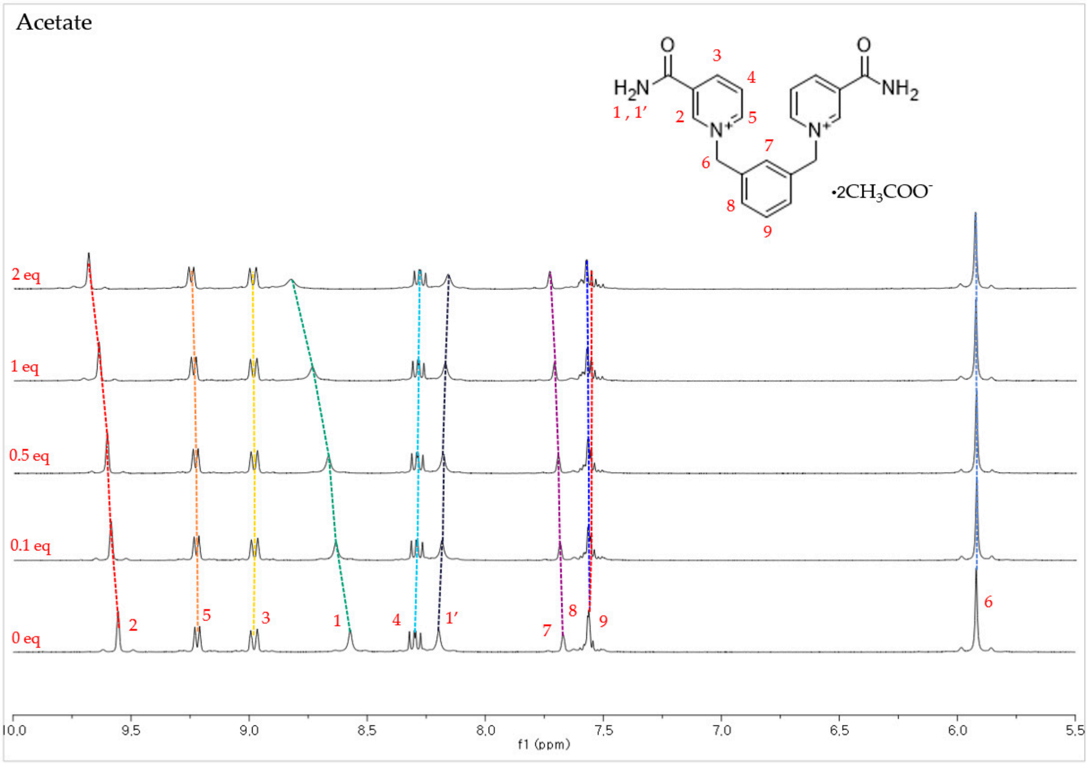

Figure S7. Acetate titration to *bis-meta*-NICO-PF_6 in DMSO-d_6.

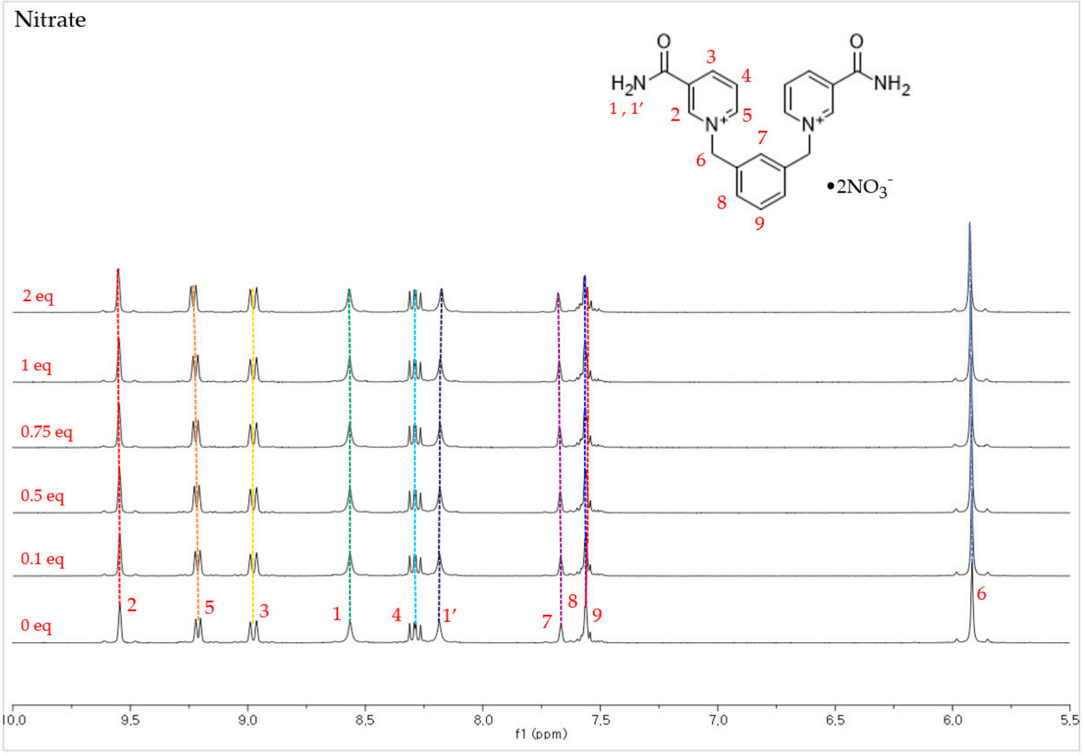

Figure S8. Nitrate titration to *bis-meta*-NICO-PF_6 in DMSO-d_6.

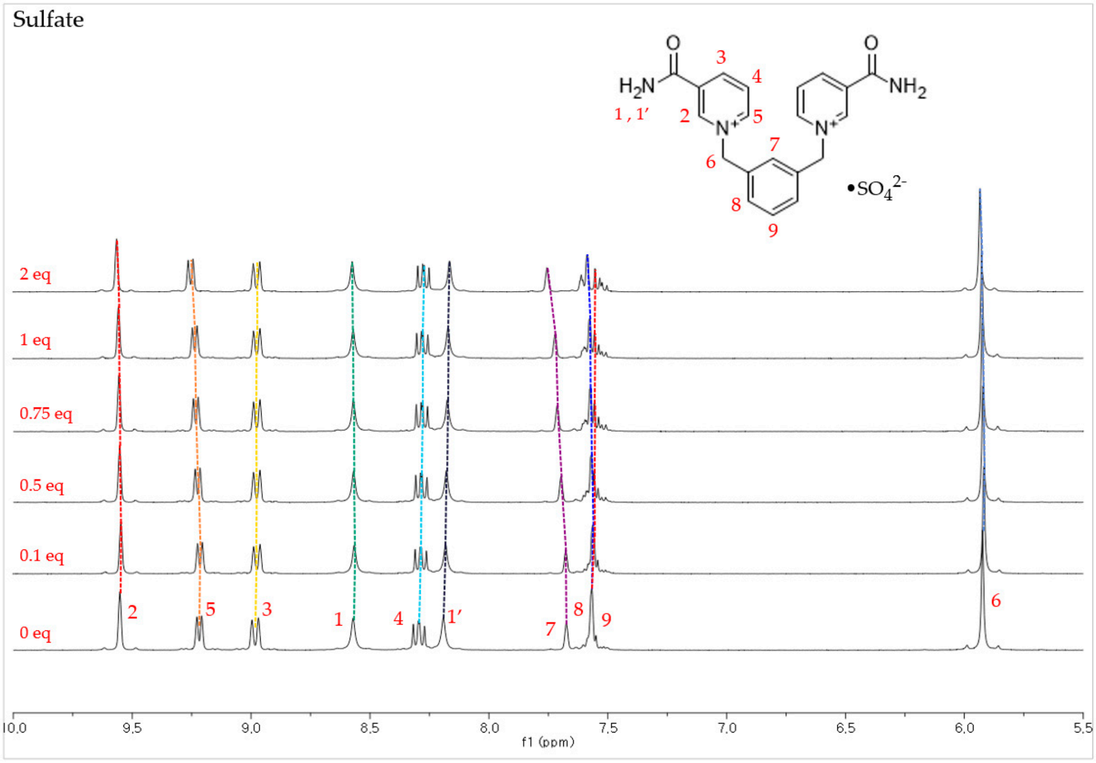

Figure S9. Sulfate titration to *bis-meta*-NICO-PF_6 in DMSO-d_6.

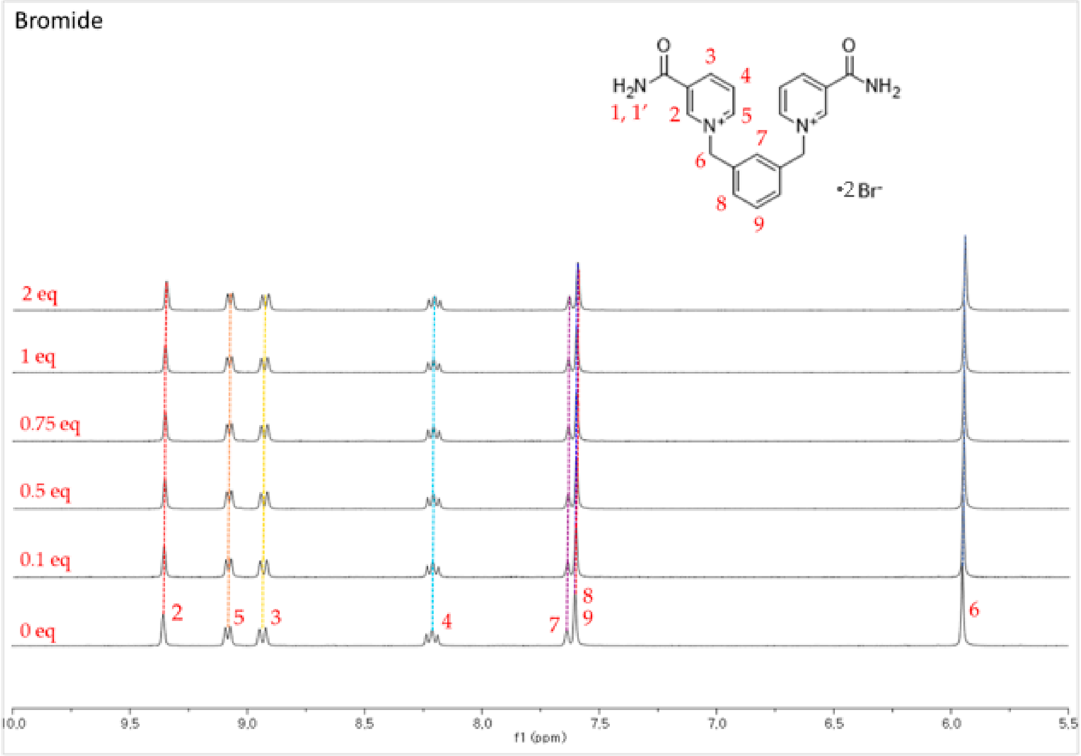

Figure S10. Bromide titration to *bis-meta*-NICO-PF_6 in DMSO-d_6.

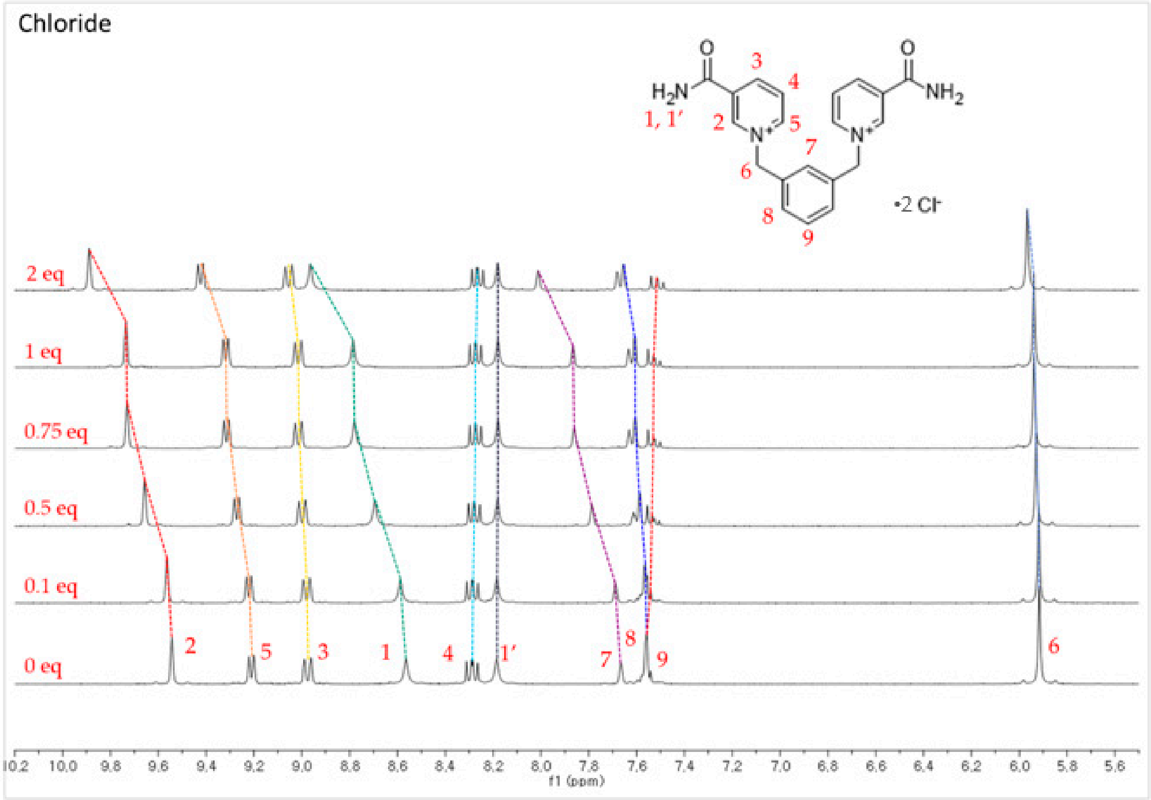

Figure S11. Chloride titration to *bis-meta*-NICO-PF_6 in DMSO-d_6.

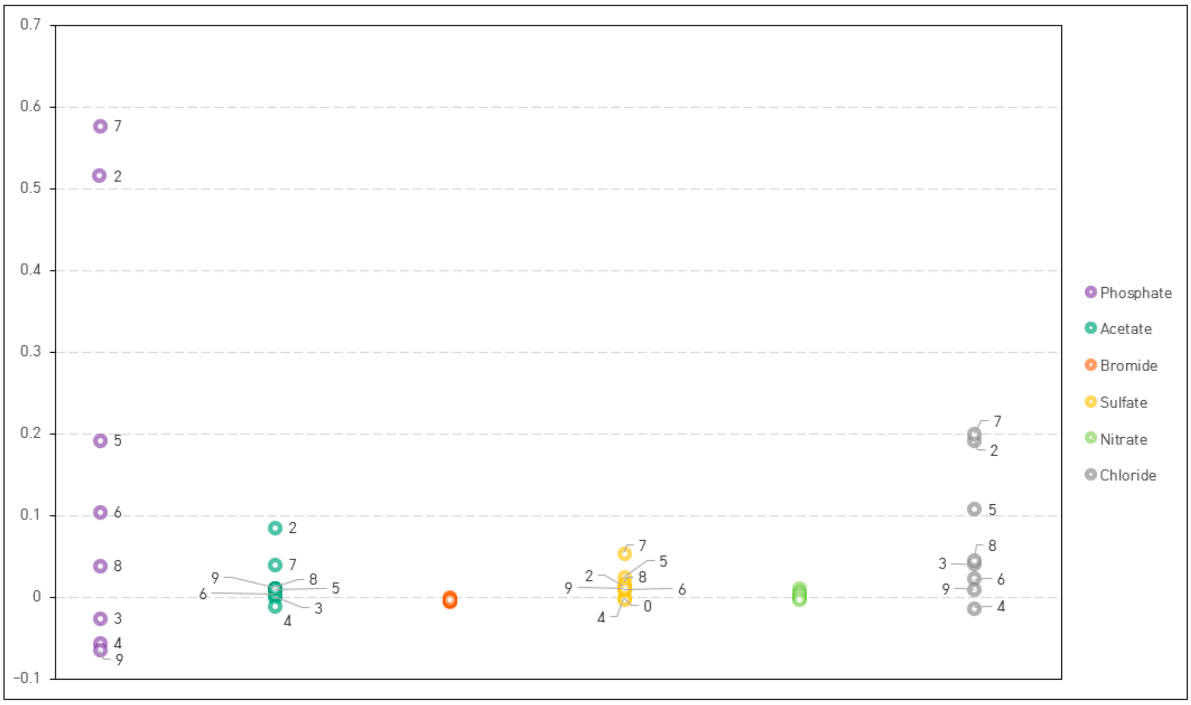

Figure S12. Chemical shift change distribution of *bis-meta*-NICO-PF_6 upon 1 equivalent of anion addition.

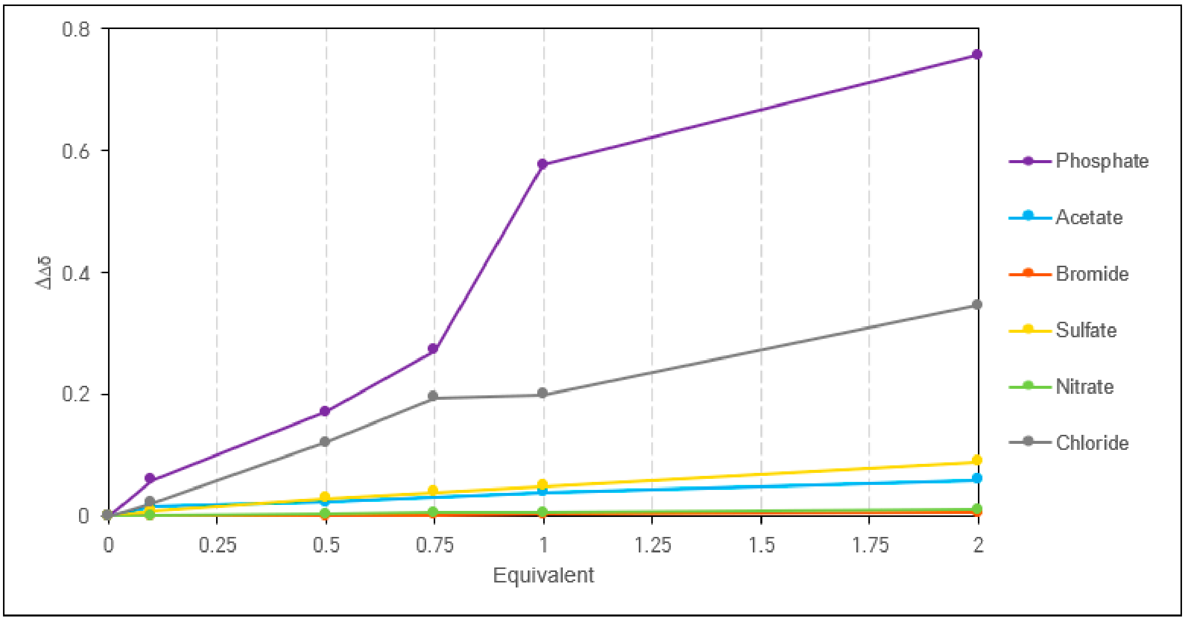

Figure S13. Chemical shift change profile of 7-H on *bis-meta*-NICO-PF_6 upon anion titration.

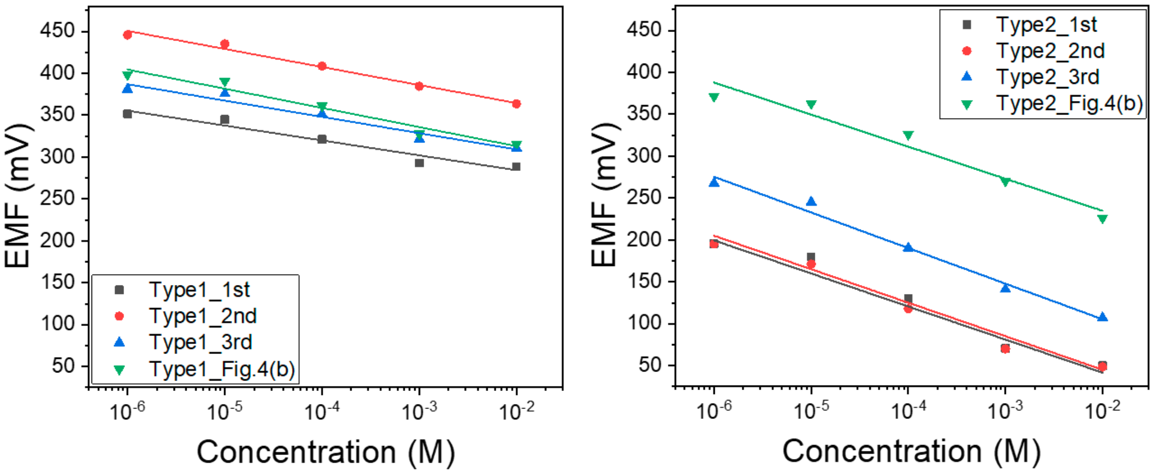

Type1	1st	2nd	3rd	Fig. 4(b)
Slope (mV/dec. of M)	-17.8	-21.6	-19.4	-22.9

Type2	1st	2nd	3rd	Fig. 4(b)
Slope (mV/dec. of M)	-40	-39.5	-42.5	-38.3

Figure S14. Type 1 and Type 2 ISE reproducibility across production batches.

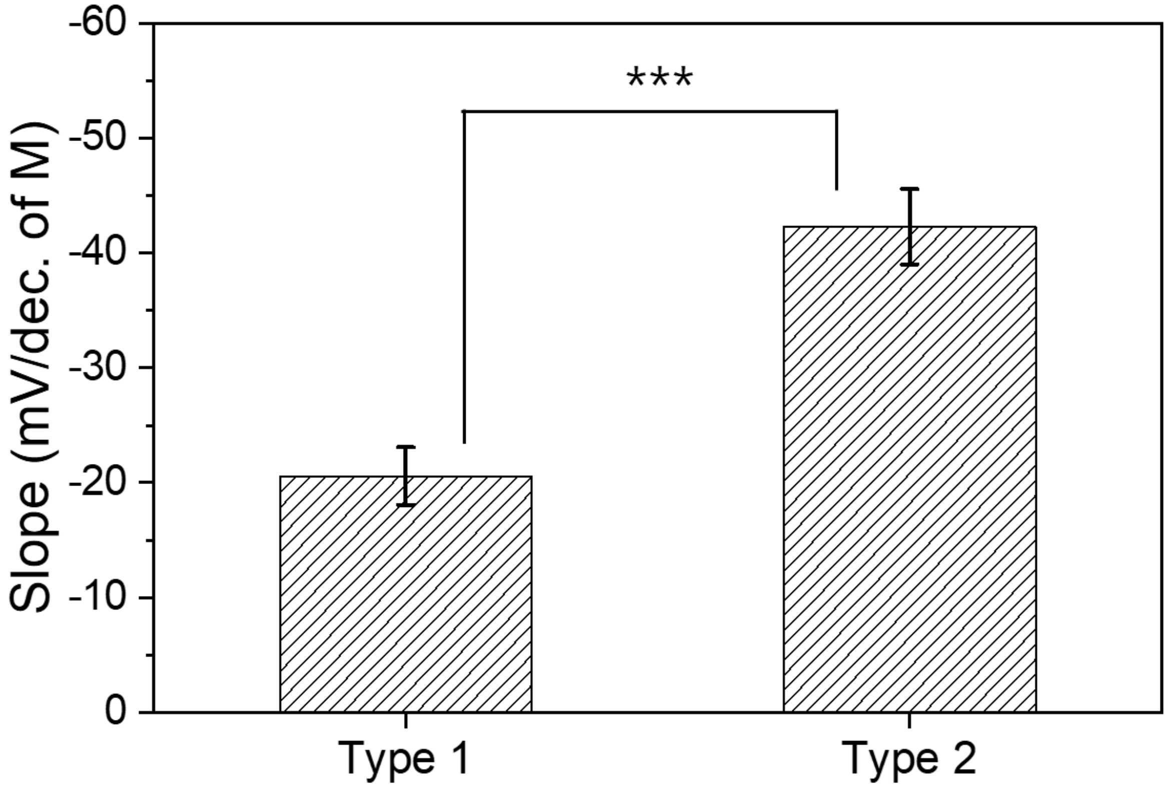

Figure S15. Slope comparison of Type 1 and Type 2 during five consecutive days. (The p-value is determined by the independent sample T verification, and the asterisk denotes the p-value, which is ***, $p < 0.001$.)
